# Src Tyrosine Kinase Inhibitors: New Perspectives on Their Immune, Antiviral, and Senotherapeutic Potential

**DOI:** 10.3389/fphar.2019.01011

**Published:** 2019-09-18

**Authors:** José Rivera-Torres, Esther San José

**Affiliations:** Department of Pharmacy, Biotechnology, Nutrition, Optics and Optometry, Faculty of Biomedical and Health Sciences, Universidad Europea de Madrid. Madrid, Spain

**Keywords:** Src tyrosine kinase inhibitors, dasatinib, immunotherapy, LGLs, γδ T cells, senescence, senolytics, progeria

## Abstract

Deregulated activity of the Src tyrosine kinases leads to malignant transformation. Since the FDA approval of the tyrosine kinase inhibitor, imatinib, in 2001 for the treatment of chronic myeloid leukemia (CML), the number of these inhibitors together with Src tyrosine kinase inhibitors (STKIs) has increased notably due to their beneficial effects. Dasatinib, a second-generation STKI inhibitor widely studied, proved high efficiency in CML patients resistant to imatinib. In the last decade STKIs have also been implicated and showed therapeutic potential for the treatment of diverse pathologies other than cancer. In this regard, we review the properties of STKIs, dasatinib in particular, including its immunomodulatory role. Similarly, the potential benefits, adverse effects, and safety concerns of these inhibitors regarding viral infections are considered. Moreover, since life expectancy has increased in the last decades accompanied by age-related morbidity, the reduction of undesirable effects associated to aging has become a powerful therapeutic target. Here, we comment on the ability of STKIs to alleviate age-associated physical dysfunction and their potential impact in the clinic.

## Introduction

Src is the prototypical member of a of nonreceptor protein–tyrosine kinases family, which in humans is composed of 11 members (reviewed in Manning et al. (2002). This family has been involved in a wide variety of essential functions to sustain cellular homeostasis where they regulate cell cycle progression, motility, proliferation, differentiation, and survival, among other cellular processes (Roskoski, 2015). As a consequence, their deregulated activity has been linked to malignant transformation, and small tyrosine kinase inhibitors have been indicated for the treatment of certain blood malignancies, including chronic myeloid leukemia (CML).

The tyrosine kinase inhibitor dasatinib demonstrated high efficacy in CML patients resistant to imatinib treatment (Weisberg et al., 2007) by targeting several Src kinases involved in the activation of the immune system (Blake et al., 2008). Importantly, emerging data from basic research and clinical trials suggest a wider potential role, for Src tyrosine kinase inhibitors (STKIs) in general and dasatinib in particular, beyond its antitumoral effect. Next, we review the novel perspectives of these inhibitors as immunotherapeutic, antiviral, and geroprotective drugs.

## Immunotherapy

### Dasatinib as Immunosuppressor

Ligand binding to TCR/CD3 complex activates diverse Src kinase family members, leading to T-cell activation. Patients with CML showing aberrant expression of the ABL-BCR Src kinase are efficiently treated with dasatinib, a broader and higher specific pan-Src kinase inhibitor (Das et al., 2006).

In fact, proteomic analyses revealed that dasatinib bound to 30 different kinases (Bantscheff et al., 2007; Rix et al., 2007) in contrast to other tyrosine kinase inhibitors such as imatinib or nilotinib, which showed a more restrictive target. These differences in the binding profile correlated with key events in T-cell activation (TCR signaling, expression of activation markers, cytokine production, and proliferation) exclusively inhibited by dasatinib.

Furthermore, the inhibitory effects of dasatinib on natural killer (NK) (Blake et al., 2008), CD4^+^, and naive T cells (Weichsel et al., 2008) suggest a potential immunomodulatory role directly targeting the TCR/CD3 complex. The possibility of combining dasatinib with other immunosuppressive agents, such as CsA and rapamycin, could represent a new way to treat CML patients and other immune-related diseases where the immune system is activated. In fact, it has been proposed very recently the use of dasatinib as a pharmacologic on/off switch for CAR T cells, an adoptive immunotherapy based on the expression of chimeric antigen receptors against cancer (Mestermann et al., 2019). This therapy has been associated with the emergence of cytokine release syndrome. Therefore, blocking Lck, as dasatinib does, could be an excellent and specific treatment for those patients (Mestermann et al., 2019)

Nevertheless, dasatinib has demonstrated to be safe and efficient upon treatment of CML patients, although side effects, such as myelosuppression, bleeding, fluid retention (Brave et al., 2008), body weight loss, severe pleural effusions, or increased risk of infections (Sillaber et al., 2009), have been reported. These adverse effects could be explained by the wider targets of dasatinib (de Lavallade et al., 2008), but the commonest are mostly moderate and usually self-limited or easy to control (Steegmann et al., 2016).

### Unexpected Effect of Dasatinib in Lymphocytosis

Monoclonal and oligoclonal expansions of LGLs, known as lymphocytosis, have been linked with a positive immune effect on tumor surveillance (Raitakari et al., 2000; Epling-Burnette et al., 2007). Large Granular Lymphocytes, encompassing CD8^+^ T lymphocytes (CD3^+^/CD8^+^effector memory cells) and NK cells (CD3^−^CD16/CD56^+^), represent 10% to 15% of the peripheral blood mononuclear cells (PBMCs).

Ten years ago, a pioneering study described a significant expansion of LGLs in PBMCs of a group of CML patients treated with dasatinib (Mustjoki et al., 2009). This unexpected outcome was further confirmed by others (Kim et al., 2009; Nagata et al., 2010; Lee et al., 2011; Tanaka et al., 2012) and correlated with patient’s good prognosis. Although the percentage of LGLs shifts from 27% to 73% depending on the study (reviewed in (Qiu et al., 2014)), generally the effect was dose-dependent, and importantly, none of the other STKIs demonstrated a similar ability.

The expansion of dasatinib-dependent LGLs could lead to a long-term cure in CML patients. In fact, the presence of LGLs at the diagnosis phase was increased during treatment (Kreutzman et al., 2010; Kreutzman et al., 2011; Tanaka et al., 2012) and accompanied by a deep molecular response, by measuring *ABL-BCR* transcript (Hughes et al., 2017). Furthermore, a case report showed extended lymphocytosis up to 2.4 years after treatment cessation accompanied by presence of memory and effector Cytotoxic T Lymphocytes and NK cells (Jo et al., 2018).

On the other hand, studies with allogenic stem cell–transplanted patients suffering from various malignant hematological diseases confirmed the emergence of NK cells together with γδ T cells and favored patient prognosis (de Witte et al., 2018). In fact, it has been reported that LGL^+^ population in dasatinib-treated CML patients had more TCR δ rearrangements compared with LGL^−^ group (90% vs 10%). These rearrangements were specifically of γδ T cells (Kreutzman et al., 2010). Analyses of a large number of human tumor samples, seeking biomarkers of overall survival outcomes, revealed the key role of infiltrated γδ T cells (Gentles et al., 2015). Evidences of the important role of γδ T cells as antitumoral agents are increasingly growing (for review, see Scheper et al., 2013; Halim et al., 2017). Moreover, their ability to eliminate minimal residual disease in pediatric CML patients with some kind of disorders (malignant or not) was shown (Airoldi et al., 2015). Many clinical trials stimulate γδ T cells with aminobisphosphonates (zoledronate in combination with other compounds) or have applied adoptive transfer of enriched specific γδ T cells (Fisher et al., 2014) to increase their antitumoral effect. The possibility to combine dasatinib with other compounds that promote the action of γδ T cells could be a new approach to broad and improve the possibilities of CML patients’ treatments.

### Immune Activation Considerations

The different outcomes of dasatinib-treated patients described above in relation to the immunosuppressive and immune-activation effects are apparently opposed, but the clinical results are indisputable. Patients with CML treated with dasatinib have better prognosis due to the presence of immune cells that protect them from a relapse. Several questions arise from these studies as to why only a group of CML patients have lymphocytosis. Therefore, an exhaustive follow-up of the patients should be carried out to elucidate the reasons underneath.

Dasatinib is a multikinase inhibitor molecule of second generation with unique characteristics compared to other STKIs that acts not only on Src family kinases, but also against c-kit, EPHA2, and PDGF receptor. This mechanism of action could confer dasatinib the ability of increased lymphocytosis and activation of the immune system, therefore conferring patients a better prognosis.

## Antiviral Effects

The complexity and redundancy of Src kinases signaling pathways provide a broad window where STKIs can be used. Currently, the field of viral infection is attracting special interest and intense research aimed at understanding and implementing their potential benefits as therapeutics.

The understanding and deciphering of mechanisms through which STKIs might work as antiviral drugs are growing. For instance, dasatinib and saracatinib have demonstrated effectiveness in modulating the life cycle of dengue virus serotype 2 at viral RNA replication by targeting Fyn kinase *in vitro* (de Wispelaere et al., 2013). Additionally, dasatinib demonstrated cooperative effect *in vitro* in combination with the viral entry inhibitor sofosbuvir on hepatitis C virus infection (Xiao et al., 2015).

Regarding the mechanisms involved in blocking HIV-1 infection, the picture is even more complex where diverse targeting has been proposed. Dasatinib was reported to control viral replication *in vitro* through negative regulation of T-cell proliferation, activation, and cytokine production, due to the inhibition of Lck kinase (Blake et al., 2008). Next, its antiviral mechanism, like imatinib and nilotinib, performed in primary and cell lines, was based on its ability to inhibit kinases involved in the virus-cell fusion process, while cell viability and viral receptor expression, required for fusion, remained unaffected. Dasatinib treatment blocked the viral entry at the hemifusion step (Harmon et al., 2010). In contrast, a study investigating whether STKIs—including dasatinib—exerted their inhibitory effect on HIV-1 infection through the interaction between c-SRC and its ligand PTK2B concluded that multiple postentry steps played a major role in blocking HIV-1 infection (McCarthy et al., 2016).

Recent studies in HIV-infected dasatinib-treated PBMCs, from healthy donors, demonstrated that the antiviral effect of dasatinib was exerted not at the viral fusion and entry step as proposed (Harmon et al., 2010) but downstream, targeting the cellular restriction factor SAMHD1 for dephosphorylation (Bermejo et al., 2016).

### Antiviral Considerations

The promising results reported on the potential use of STKI to fight against viral infection, in particular HIV-1, have paved the way for an exciting field of research. However, we consider that several issues should be addressed before moving ahead, such as by using humanized preclinical models to optimize both the treatment scheme and the safety of a combined and lifelong therapy based on STKIs and antiretroviral drugs. The mechanisms to be addressed include shifts in apoptosis and/or signaling pathways, susceptibility to opportunistic infections, and the ability to trigger reactivation of other viral coinfections. In fact, it has been reported that some dasatinib-treated CML patients experienced symptomatic cytomegalovirus reactivation (Kreutzman et al., 2011).

A recent detailed review of the potential use of STKIs as well STKI-related safety concerns in HIV-1 infection is recommended for interested readers (Coiras et al., 2017).

## Senotherapeutics

Aging constitutes one of the major risks of morbidity and mortality, with senescence being one of the well-established aging hallmarks (Lopez-Otin et al., 2013; McHugh and Gil, 2018). Cell senescence defines an irreversible cell-cycle arrest accompanied by phenotypic changes including resistance to apoptosis and production of senescent-associated secreted phenotype (Coppe et al., 2008). Although this cellular process is beneficial under healthy physiological conditions, the accumulation of senescent cells underlined tissue damage in preclinical models (Kirkland and Tchkonia, 2015). Moreover, the sole presence of these cells is sufficient to produce physical dysfunction and reduced survival rate and life expectancy (Xu et al., 2018). Hence, the proof of concept that elimination of senescent cells delayed age-associated disorders (Baker et al., 2011) established a causal effect between cellular senescence and aging.

### Senotherapeutic Considerations and Perspectives of STKIs

Because of the detrimental effect of senescent cells in a plethora of age-related disorders such as atherosclerosis, cachexia, and sarcopenia (McHugh and Gil, 2018), their selective elimination represents an important target for therapeutics development. Among the diverse approaches, senolytics constitute one of the most promising antisenescence therapies. In support of that, and thanks to the use of navitoclax, a first-generation senolytic targeting antiapoptotic proteins, the role of senescent astrocytes and microglia in the etiology of neurodegenerative diseases has been recently stablished (Bussian et al., 2018).

Similarly, STKIs have also been proposed as potential therapeutic agents to fight osteoporosis, another major health problem in the elderly. Since Src kinase is expressed and required for the development of the ruffled border of the osteoclast, the STKI saracatinib has been shown to play a role on bone turnover (Hannon et al., 2010). More recently, the anabolic and antiresorption effects of dasatinib on human osteoblast and osteoclast differentiation and function were reported (Garcia-Gomez et al., 2012).

Several reports have also established the importance of STKIs as promising agents against a plethora of fibrotic, chronic, and age-related diseases in several tissues (reviewed by Wang and Zhuang (2017). Specifically, treatment of epithelial cells and renal interstitial fibroblasts with dasatinib demonstrated suppression of renal fibrosis by targeting Hck, the sole member of the Src kinase family being upregulated in the kidney from renal-transplanted patients (Wei et al., 2017). The authors confirmed their results in a mouse model of renal interstitial fibrosis based on reduction in fibrotic markers (collagen, vimentin, and MMP-2, among others) and proinflammatory cytokines. Similarly, nintedanib (Awasthi and Schwarz, 2015), PP2 (Mima et al., 2011; Taniguchi et al., 2013; Wu et al., 2015), SU6656 (Das et al., 2016), and bosutinib (Sweeney et al., 2008; Elliott et al., 2011) demonstrated effectiveness as potential chronic kidney disease therapeutics.

Combination of senolytics, targeting different pathways, might expand the range of target cells. Specifically, the first combination reported so far (dasatinib plus quercetin [D+Q]) increased the ablation of senescent cells, compared to the effect demonstrated independently (Zhu et al., 2015; Xu et al., 2018). In fact, this combination is currently being tested in clinical trials. NCT02874989 trial aimed to eliminate proinflammatory cells in patients suffering from idiopathic pulmonary fibrosis (Kirkland and Tchkonia, 2017; Katsuumi et al., 2018; Justice et al., 2019). The ability of D+Q is also being tested in an ongoing clinical trial (NCT02652052), which aims to evaluate age-related changes in transplanted survivors.

Likewise, senescence also represents one of the features of an extremely rare and segmental disease known as Hutchinson–Gilford progeria syndrome (HGPS) (Opresko et al., 2002; Kudlow et al., 2007). Patients with HGPS expressing progerin, an unprocessed nuclear lamin A–dominant mutant, show aging features typical of the elderly and undergo accelerated aging, leading to death caused by cardiovascular disease at an average age of 13.5 years (Trigueros-Motos et al., 2011)

Progerin accelerates cellular stressors, including DNA damage and genomic instability (Gonzalo and Kreienkamp, 2015), upregulation of p53 signaling pathway (Varela et al., 2005; Benson et al., 2010; von Muhlinen et al., 2018), and mitochondrial dysfunction (Rivera-Torres et al., 2013), among others. These stimuli trigger senescence in mouse model of the disease (Liu et al., 2013) as well as in cell lines isolated from HGPS patients (Benson et al., 2010; Wheaton et al., 2017).

Recent studies have demonstrated cardiac electrical defects both in a progeroid mouse model as well as in HGPS patients (Rivera-Torres et al., 2016; Filgueiras-Rama et al., 2018). Moreover, the progeroid mouse model showed a sharp decrease in vascular smooth muscle cells (VSMCs) in the medial layer of the aortic arch (Osorio et al., 2011). Progeroid aortic VSMC had an impaired capacity to inhibit vascular calcification as a result of mitochondrial dysfunction, leading to excessive vascular calcification (Villa-Bellosta et al., 2013). Interestingly, D+Q treatment decreased senescence cell markers in the medial layer of the vasculature, although its effects on calcification have not been analyzed yet (Roos et al., 2016).

Considering the complete absence of any treatment for HGPS patients, and in agreement with a recent report suggesting the feasible role of quercetin in attenuating cellular senescence in HGPS (Geng et al., 2018), it is interesting to speculate whether the senolytic approach could help ameliorate the aged phenotype of progeroid preclinical models and, if so, be transferred to clinics.

## Concluding Remarks

Increasing evidence reinforces the potential of STKIs, with dasatinib being the most widely studied, as therapeutics in biomedical fields other than cancer. Herein, we have reviewed their novel role and their future perspectives in immunotherapy, viral infections, and geroprotection.

Despite the reported efficacy and safety of dasatinib, certain questions remained to be addressed before results can be translated from preclinical studies into clinics. Although its long-term effect has been studied as immunotherapeutic, it needs to be implemented for other pathologies. Overall, a further characterization of the mechanisms and functions underlying the use of STKIs in diverse contexts looks essential in order to design specific therapies. Additionally, the identification of potential off-target effects raised during prolonged treatments is required. Such treatments should be tested in order to clearly define the link with an increase in healthy life expectancy as well as in ameliorating the pathology.

The use of dasatinib as immunomodulatory agent has opened a broader window for the treatment of diseases other than CML. On the one hand, it could be possible to use it as an immunosuppressor as recently it has been shown for CAR T-cell immunotherapy or even in autoimmune diseases where a profound deregulation of the immune system takes place. On the other hand, and due to its immune activation effect, the potential immune activation due to the emergence of lymphocyte changes in number and a favorable discontinuation of dasatinib is an area of growing interest, due to prolonged treatments and due to financial issues. The achievement of deep molecular responses together with a profound immunosurveillance in some dasatinib-treated patients demands looking for specific predictive biomarkers to asses which patients could interrupt the treatment without undergoing decay. Several studies have shown up the analysis of putative biomarkers as CTL-A4, TFG-β, PDMPs (platelet-derived microparticles), and PD-1, but more clinical trials should be done to confirm these data (Hughes et al., 2017; Nomura et al., 2019).

Regarding the antiviral effect of dasatinib, it is important to mention that the role of the cellular restriction factor SAMHD1 is not restricted to RNA viruses (Ballana and Este, 2015) but to DNA viruses including poxviruses (Hollenbaugh et al., 2013), herpes simplex 1 (Kim et al., 2013), and hepatitis B (Chen et al., 2014; Jeong et al., 2016; Sommer et al., 2016). Hence, it suggests a potential role of dasatinib in viral infections other than RNA as therapeutic.

The use of dasatinib as senolytic avoids the side effects upon accumulation of senescent cells in specific tissues in preclinical model. However, a recent report casts doubts on its beneficial effects to clear senescent cells upon liver cancer chemotherapy (Kovacovicova et al., 2018).

In summary, we consider that translating outcomes obtained by using preclinical models into clinics is highly challenging and that further research is required to shed light on the precise role of STKIs, especially for the senotherapeutic field, which is still in its infancy.

## Author Contributions

JR-T and EM conceived and wrote the manuscript. Both authors contributed equally.

## Funding

Publication fee was supported by Universidad Europea de Madrid.

## Conflict of Interest Statement

The authors declare that the research was conducted in the absence of any commercial or financial relationships that could be construed as a potential conflict of interest.
